# Segregation and Life Satisfaction

**DOI:** 10.3389/fpsyg.2020.604194

**Published:** 2021-02-05

**Authors:** Rodrigo Montero, Miguel Vargas, Diego Vásquez

**Affiliations:** ^1^Facultad de Economía y Negocios, Universidad Andrés Bello, Santiago, Chile; ^2^Observatorio Social, Ministerio de Desarrollo Social, Santiago, Chile

**Keywords:** subjective wellbeing, quality of life, segregation, inequality, social capital, small area estimations

## Abstract

Our aim is to cast light on socioeconomic residential segregation effects on life satisfaction (LS). In order to test our hypothesis, we use survey data from Chile (Casen) for the years 2011 and 2013. We use the Duncan Index to measure segregation based on income at the municipality level for 324 municipalities. LS is obtained from the CASEN survey, which considers a question about self-reported well-being. Segregation’s impact upon LS is not clear at first glance. On one hand, there is evidence telling that segregation’s consequences are negative due to the spatial concentration of poverty and all the woes related to it. On the other hand, segregation would have positive effects because people may feel stress, unhappiness, and alienation when comparing themselves to better-off households. Additionally, there is previous evidence regarding the fact that people prefer to neighbor people of a similar socioeconomic background. Hence, an empirical test is needed. In order to implement it, we should deal with two problems, first, the survey limited statistical significance at the municipal level, hence we use the small area estimation (SAE) methodology to improve the estimations’ statistic properties, and second, the double causality between segregation and LS; to deal with the latter, we include lagged LS as a regressor. Our findings indicate that socioeconomic segregation has a positive effect on LS. This result is robust to different econometric specifications.

## Introduction

What is the effect of socioeconomic residential segregation on subjective life satisfaction (LS)? This is the research question of the present investigation. The relevance of this question lies, mainly, in the following reasons. First, LS is an important component of individuals’ well-being, and therefore, it should be analyzed for exploring appropriate policies to protect and improve well-being (Ruggeri et al., 2020). The latter demands to have an understanding of what the drivers are behind LS, and according to the literature, there is a nexus between LS and residential satisfaction (Ibem and Amole, 2013). As a matter of fact, LS is an aggregate satisfaction in the different life domains (Van Praag et al., 2003). These include social relationships, education, and housing (Argyle, 2001). Previous investigations have identified a link between housing environment and overall well-being (LS is part of the overall well-being) (Bovaird and Elke, 2003; Park, 2006). The type of housing and quality of surrounding environment have a significant influence on people’s overall well-being (Bashir, 2002; Cazacova et al., 2010; Theriault et al., 2010). Hence, previous investigations suggest that households’ LS is influenced by their satisfaction with housing environment, which is composed, among others, of the location and socioeconomic characteristic of their neighborhoods (Ibem and Amole, 2013).

Second, the answer to this question is not clear at first sight. Previous investigations have pointed out the pernicious effects of segregation on a wide range of individuals’ economic, health, and educational outcomes (Jencks and Mayer, 1990; Brooks-Gunn et al., 1993; Cutler and Glaeser, 1997; Leventhal and Brooks-Gunn, 2000; Fabio et al., 2009; Jargowsky, 2014; Chetty et al., 2016 and Sampson et al., 2002). Regarding mental health, Ludwig et al. (2012) find that neighborhoods have effects on subjective well-being (SWB) (once again, LS is one of the components of well-being) and mental health; hence, people living in high-poverty neighborhoods will improve their LS if they move to lower-poverty neighborhoods. In the same line, Bittencourt et al. (2020) explore how differences in scale, geography, and race interact with segregation and the impact that these interactions have on the access to cities’ amenities by public transport, finding that accessibility varies across the socio-spatial structure. Gibbons et al. (2020) found that segregation is associated with clusters of poor health households albeit the final effect depends on races and ethnicities being poor—afro descent households the most affected group. Jimmy et al. (2019) describe the association between residential segregation and quality of life in the city of Nairobi. They found a positive correlation between symbolic integration, safety, and quality of life related to housing in poor neighborhoods but a negative correlation of these variables in gated communities. However, evidence about segregation’s positive effects on individuals’ LS has been found as well. For instance, racial homogeneity is related to lower rates of psychosis, suicide, common mental disorders, and mortality (Okulicz-Kozaryn, 2019). Besides, segregated neighborhoods may induce people to a greater sense of belongingness, which induces social cohesion, trust, participation, mutual support, collective action, and social capital (Alesina and La Ferrara, 2000; Luttmer, 2001; Luttmer and Singhal, 2008; Stafford et al., 2010). Additionally, the poor may feel stress, unhappiness, and alienation when comparing themselves to better-off people (Davis, 1988), and there is previous evidence regarding the fact that, on average, adult people prefer to neighbor people of a similar socioeconomic background (Luttmer, 2005). Segregation, particularly when the segregated group corresponds to better-off members of society, produces a greater level of labor productivity (Díaz et al., 2020). Social interactions within neighborhoods are a significant device to find a job among peers; hence, they boost labor market matchings. Segregated neighborhoods may provide consumption benefits due to the fact that individuals of similar income and preferences tend to consume similar goods and similar local amenities (Cheshire, 2007). Moving grown-ups and adolescents from poor to richer neighborhoods has a negative impact maybe due to disruption effects (Chetty et al., 2016). In the same line, it has been found that Whites, Afro Americans, and Hispanics are happier among their own race (Okulicz-Kozaryn, 2019). From the mental health point of view, there are several investigations pointing out the possible positive effects of segregation (Wilkinson and Pickett, 2008; Stafford et al., 2010; Shaw et al., 2012). Particularly, the focus is on what has been called “the ethnic density hypothesis,” which suggests that living in neighborhoods of higher own-group density may be protective for mental health. This effect may operate through a buffering effect for individuals living in high own-group density areas through improved social networks or due to the reduction of the frequency of negative experiences like racism (Das-Munshi et al., 2010). Possible mechanisms behind protective effects of own-group density on mental health have to do with enhanced social support, mitigated negative attitudes from other groups, positive identity, and higher self-evaluation. In this sense, own-group density may promote resilience by providing appropriate social support with which to resist the psychological stresses due to negative attitudes coming from different socioeconomic groups. Living in areas with people more like oneself may reinforce one’s own identity and allow an individual to view himself or herself with higher self-esteem, as it is widely acknowledged that identity and self-evaluation are both self-determined and shaped by the definitions of others, which may incorporate the perceived views of one’s local community (Jenkins, 2004; Shaw et al., 2012). Similar results are found in Chile as discrimination has a negative impact on psychological well-being, and collective identity has a positive one. Consequently, promoting the sense of belongingness and the own-group self-esteem would encourage mental health (García et al., 2017).

To answer this question, we conduct an econometric analysis using the Chilean National Socio-Economic Characterization Survey (Casen) for the years 2011 and 2013. In 2011 and 2013, CASEN includes a question about LS. As a dependent variable, we use the municipality average level of LS, and as regressors, those controls that theory and previous research have identified as determinants of LS plus municipalities segregation, which have been measured using the Duncan Index. Our main result is that socioeconomic segregation has a positive and significant effect on LS. We run different specifications: ordinary least square (OLS), OLS including lagged LS, and first difference to control for non-observed fixed effects. In all these specifications, the socioeconomic segregation estimated parameter is positive and significant. To the best of our knowledge, this is the first investigation that searches to cast light on income base segregation effects on LS in a Latin American country.

It is important to stress here the difference between a municipality socioeconomic composition and its segregation. A municipality may be poor, for instance, if 80% of the population is poor, but not necessarily segregated if every block has the same 80% of poor households, but it will be segregated if it has the same 80% of poor people but some blocks concentrate the total amount of poor inhabitants. In the present investigation, the focus is on segregation. Residential segregation is a multidimensional phenomenon, as it has been defined in the literature (Massey and Denton, 1988). Specifically, this article defines five dimensions: evenness, exposure, concentration, centralization, and clustering. The vast majority of investigation on this subject has been focused on evenness, and it is not very common to find an article dealing with more than one dimension, a phenomenon called hypersegregation (see, for instance, Massey and Tannen, 2015). In the present investigation, we consider as residential segregation evenness because it fixes better to the Chilean cities geographical structure and because it has been widely used and hence it is easier to make comparisons with other findings in the literature. Additionally, some dimensions, like centralization, have lost relevance. The best index to measure evenness is the Dissimilarity Index (Massey and Denton, 1988), which is the one used here. It is important to point out the fact that Chilean cities exhibit high levels of segregation, something that has been corroborated by different investigations and using different segregation measures (Lambiri and Vargas, 2011; Vargas, 2016; Garrido and Vargas, 2020).

Our result may seem controversial; however, it is important to point out the fact that well-being is a multidimensional construct that goes beyond hedonism and the pursuit of happiness or pleasurable experiences and that an informative measure of well-being must encompass both hedonic and eudaimonic aspects (Ruggeri et al., 2020), and, albeit this is an important component of well-being, because of that, it is relevant to pay attention to those factors that are behind it; in order to implement a public policy, it is crucial to know how segregation would affect the full set of dimensions that compose well-being.

Summarizing, the effort of understanding determinants of LS is important to individuals and society as a whole because it is strongly connected to people quality of life, hence it will help design public policies that contribute to improve society’s quality of life (Skevington and Böhnke, 2018; Ruggeri et al., 2020). LS offers a good measure of human progress as well because it takes into account more factors than gross domestic product (GDP) alone (Diener et al., 2013). Besides, there are several investigations that have shown that LS has objective benefits on major life domains such as health and longevity; income, productivity, and organizational behavior; and individual and social behavior (De Neve et al., 2013).

## Materials and Methods

We use the Casen survey for the years 2011 and 2013. This survey is one of the main sources of socioeconomic characterization used in Chile for public policy design and impact evaluation. This survey is taken every 2 years, and the sample design is probabilistic and stratified according to geography and population size. The sample selection is made in two stages in urban areas and in three stages in rural areas. In 2011, this survey was applied to 59,084 households; meanwhile, that in 2013, to 66,725—both in 324 municipalities. However, the statistical significance is not at municipality level but at national and regional levels and at urban and rural areas. The latter presents a challenge as we use segregation at the municipality level; therefore, we need to characterize LS at the same level. In order to deal with this problem and to improve the statistical properties of our estimations, we implement the small area estimation (SAE) methodology, which is explained in section *Small Areas Estimations*. Each municipality is divided into segments (census tracts), which are the primary sampling units.

The decision of using these two particular years is based on the fact that the Casen survey included a question about LS in 2011 and 2013. The exact question was: “Taking into account all aspects in your life: How satisfied are you with your life?” According to international standards, the answer to this question is in a scale that goes from 1, fully unsatisfied, to 10, fully satisfied.

### Small Area Estimations

As mentioned, to face the statistical limitations of Casen when the unit of analysis is more disaggregated, we implement the SAE methodology. Following this procedure, we improve the precision of our dependent variable (Molina and Rao, 2010; Ministerio de Desarrollo Social y Familia, 2013; Casas-Cordero et al., 2016; Molina, 2019).

SAEs comprise a range of alternative procedures, but given the nature of the Casen data, we will use the Fay–Herriot model, which provides estimates at the area level. This model links an indicator δ_d_ for all areas d = 1…D using information from the survey (${\hat{\delta }}_{\text{d}}^{\text{DIR}}$) and the prediction of a synthetic regression model.

The advantages of this procedure are multiple. This estimator usually improves the efficiency of the direct estimations, and regression incorporates heterogeneity not explained by the areas. Additionally, the estimator of the mean square error is stable.

The direct estimator has the form ${\hat{\delta }}_{d}^{DIR}=\delta_{d}+e_{d}$, where *e*_*d*_∼*iid*(0,ψ_*d*_), and the synthetic estimator can be written as $\delta_{d}=x_{d}^{\prime }\tilde{\beta }+u_{d}$, where $e_{d}∼iid\left(0,\sigma_{u}^{2}\right)$. In general, the model is formulated as follows:

$${\hat{\delta }}_{d}^{DIR}=x_{d}^{\prime }\tilde{\beta }+u_{d}+e_{d}$$

where $\tilde{\beta }$ is the feasible least squares:

$$\tilde{\beta }={\left(\sum_{i=1}^{D}{\hat{\gamma }}_{d}x_{d}x_{d}^{\prime }\right)}^{-1}\sum_{i=1}^{D}{\hat{\gamma }}_{d}x_{d}{\hat{\delta }}_{d}^{DIR}$$

and ${\hat{\gamma }}_{d}$ is the shrinkage factor ${\hat{\gamma }}_{d}=\frac{{\hat{\sigma }}_{u}^{2}}{{\hat{\sigma }}_{u}^{2}+\psi {}_{d}}$.

Finally, the Fay–Herriot model can be expressed as a linear combination of both estimations:

$${\tilde{\delta }}_{d}^{FH}={\hat{\gamma }}_{d}{\hat{\delta }}_{d}^{DIR}+\left(1-{\hat{\gamma }}_{d}\right)x_{d}^{\prime }\tilde{\beta }$$

The variance of the estimations will determine the weight assigned to each source of information. The smaller the variance of the direct estimate, the greater is the weight. Another thing to consider is that variances of the direct estimate are heteroscedastic. Each municipality (local area) has a different variance, which is estimated based on its standard error. In our case, this corresponds to the standard error of the average of LS by municipality. The variance of the synthetic model is homoscedastic, and the efficiency will depend on the goodness of fit achieved. We estimate ${\hat{\sigma }}_{u}^{2}$ using the Restricted Maximun Likelihood (Molina and Marhuenda, 2015).

The main objective of the SAE estimation is to fortify the direct estimates and absorb the impact of working at a small area level, especially due to the probability of being in the presence of inaccurate indicators (Polettini and Arima, 2015). To solve this problem in the LS variable, we have resorted to the use of external information, where the mission is to provide support to the data that we are faced with. Having auxiliary data with no out-of-range observations and no measurement error is vital to safeguard the good properties of this implementation (Xie et al., 2007; Ybarra and Lohr, 2008).

The variables previously described were used to make the SAE poverty estimations published by the Ministry of Social Development and Family. The selection of these variables was carried out following Casas-Cordero et al. (2016) using the stepwise procedure (Table 1). On the other hand, these data were collected from reliable administrative records that are reported periodically. The percentage of people in both private and public health systems is calculated as the number of people who attend these systems (granted by each of the relevant institutions) divided by the total number of people in each municipality (information based on population projections). The number of people in the formal employment system is provided by the Unemployment Fund Administrator (AFC), and the percentage of people living in rural conditions is provided by the National Institute of Statistics.

**TABLE 1:** Descriptive statistics of auxiliary information.

Variable	Observations	Mean	SD	Minimum	Maximum
Number of people in formal employment system	345	12,673	22,249.9	6	159,652
% of Rurality	345	0.39	0.30	0	1
% of People in private health system	345	0.05	0.06	0.00	0.44
% of People in poorest public health system	345	0.21	0.10	0.01	0.70

*Source: Census 2002, National Institute of Statistics and Administrative Records.*

Socioeconomic segregation is measured using the Dissimilarity Index. This index indicates departure from an even distribution across the space by taking the weighted mean absolute deviation of every unit’s minority proportion from the city’s minority proportion and expressing this quantity as a proportion of its theoretical maximum (James and Taeuber, 1985; Massey and Denton, 1988). This index varies between 0 and 1, and, conceptually, it represents the proportion of minority members that would have to change their area of residence to achieve an even distribution, with the number of minority members moving being expressed as a proportion of the number that would have to move under conditions of maximum segregation (Jakubs, 1977; Massey and Denton, 1988). This index has been widely used in the literature because it is very easy to calculate; it demands very few data, and it is easy to make comparisons with other studies. Additionally, this is the index with the best statistics properties to measure the segregation dimension of evenness (Massey and Denton, 1988). However, it possesses some weaknesses as well. The most important is that this index does not take into account spatial aspects of segregation such the well-known modifiable areal unit problem where the arbitrary selection spatial partition, such as census tracts, county districts, or post code areas, would generate statistical bias. Several corrections to this issue have been proposed (see, for example, O’Sullivan and Wong, 2007); however, all of them demand spatial data. The nature of our data does not allow us to undertake a spatial analysis; consequently, we will use the traditional Dissimilarity Index without spatial adjustments. Notwithstanding, it has been shown that the traditional Dissimilarity Index is highly correlated with other more sophisticated measures of evenness.

The formula of this index is as follows:

$$D=\sum_{n=1}^{n}\left(\frac{t_{i}}{p_{i}}-P/2TP\left(1-P\right)\right)$$

where *t*_i_ and *p*_i_ are the total population and minority proportion of areal unit *i*, in our case, municipalities’ segments, and *T* and *P* are the population size and minority proportion of the municipality, which is subdivided into *n* segments.

Table 2 shows descriptive statistics after implementing SAE methodology. Regarding LS, it is possible to see that it is around 7 on a scale of 1 to 10 (7.09 for 2011 and 7.47 for 2013). The segregation index, calculated with Duncan’s methodology, is around 0.7 in said period. As expected, for that period, the unemployment rate was around its natural level (between 3 and 4% in the case of Chile). As can be seen, for both years, information is available for 324 municipalities.

**TABLE 2:** Descriptive statistics.

	Casen 2011	Casen 2013
Life satisfaction	7.09	7.47
Segregation	0.68	0.69
% People in public health system	0.43	0.38
% People in private health system	0.07	0.07
Labor participation rate	0.53	0.53
Unemployment rate	0.04	0.04
% Women	0.52	0.52
Age	35.9	36.5
% Elderly	0.15	0.16
Observations	324	324

*Source: Casen survey 2011 and 2013.*

**FIGURE 1:**
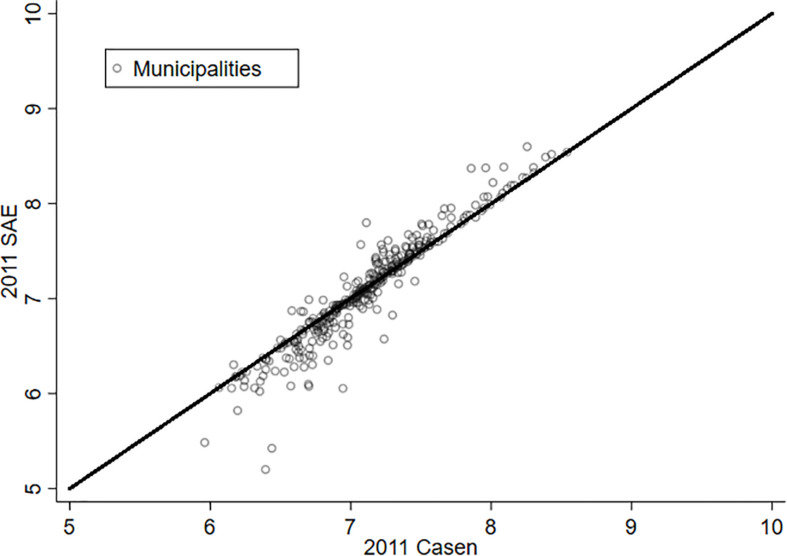
Life satisfaction and SAE, 2011.

**FIGURE 2:**
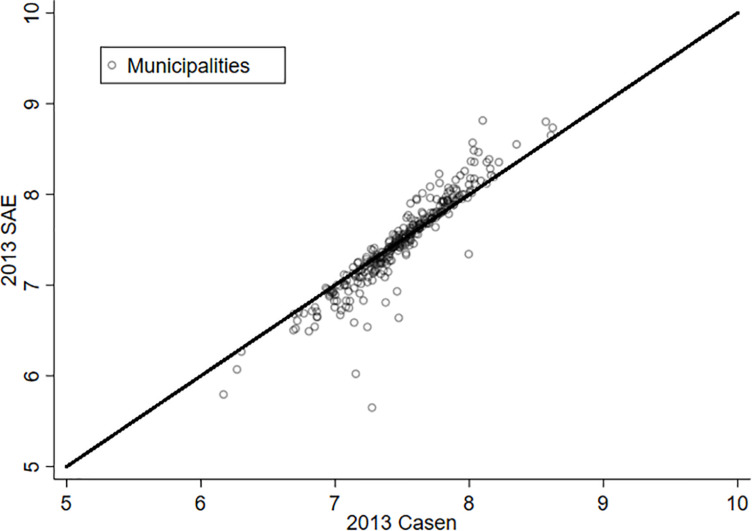
Life satisfaction and SAE, 2013.

As a way to appreciate the effect of estimating LS through the SAE methodology, Figures 1, 2 present the relationship between the original variable (X axis) and its estimation *via* SAE (Y axis) for the years 2011 and 2013. The municipalities that are on the red line (45 degrees) are those where the SAE estimate does not make a difference from the original variable. In this way, it is possible to appreciate that the majority of the municipalities experience a significant change in the variable “life satisfaction” for both years. It is also possible to notice that there are some communes that experience a substantive variation in this variable. Hence, what is learned from this exercise is that the estimation by SAE offers an improvement when working with data at the municipality level—at least for the case of Chile.

### Robustness Analysis: Regression to the Proportion

The database we have is at an aggregate level. Given this, the variables are at the average level. According to O’Connell (2000), we could have problems in the measure of average LS, since its original nature is ordinal. To solve and reaffirm the robustness of the results, a regression was performed on the transformed dependent variable of satisfaction with life. This variable represents “the percentage of people who are satisfied with their life at the communal level,” and it is a variable that is in the interval [0,1].

In addition, SAE estimates are subject to additional variance treatments, since an endogeneity problem arises: The definition of variance of a proportion depends on the estimated proportion.

Jiang et al. (2001) proposed a transformation to the dependent variable when it is a proportion and in this way avoids the endogeneity of variance problems. The transformation is arcosenic and is defined as $\arcsin\left(\sqrt{LS_{i}}\right)$, while the variance estimate associated with this transformation takes the form $\frac{deff_{i}}{4n}$, where *deff* is the design effect associated with each area, in our case, municipalities, and *4n* is four times the size of the sample associated with each area.

Considering these estimations represents a challenge that allows verifying the robustness of the available data. The following is the regression of the synthetic estimate associated with the proportion of people satisfied with their life, which takes the value 1 when the person answers 6 or more to the question described previously and 0 otherwise (Table 3).

**TABLE 3:** Proportion analysis.

	Model (1)	Model (2)
Log (monetary household income)	0.0570**	0.0594**
	(0.0275)	(0.0244)
Labor participation rate	−0.178**	−0.176**
	(0.0886)	(0.0746)
Unemployment rate	−0.0790	−0.0765
	(0.184)	(0.163)
% women	0.0165	−0.00188
	(0.162)	(0.142)
Age	−0.0125	−0.0121
	(0.0105)	(0.00892)
Age squared	0.000139	0.000130
	(0.000146)	(0.000124)
% Elderly	−0.183	−0.162
	(0.296)	(0.259)
% People in public health system	−0.0412	−0.0405
	(0.0403)	(0.0367)
10/40 Index	−9.04e-05	−0.000139
	(0.000326)	(0.000293)
% People in private health system	0.00598	−0.00279
	(0.0844)	(0.0755)
Segregation	0.0634***	0.0610***
	(0.0200)	(0.0180)
Constant	0.345	0.326
	(0.411)	(0.372)
Observations	310	310
R-squared	0.096	0.116

Robust standard errors in parentheses.

***p < 0.01; **p < 0.05; *p < 0.1.

We have chosen to follow model 2. This model incorporates the SAE poverty rate as a predictor. The inclusion of regions as control variables does not allow the percentage of rurality to remain significant, but the recommendations aim to maintain the variable even when it loses significance.

**FIGURE 3:**
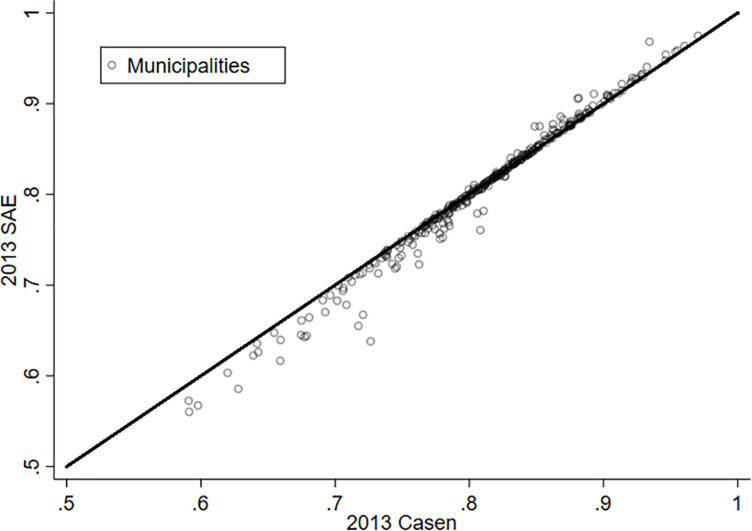
Life satisfaction and SAE for the proportion of people who are satisfied with their lives, 2013.

Finally, it should be noted that when we consider SAE technology to improve the characteristics of the LS variable, in addition to the considerations applied to the direct variance estimates, they allow us to conclude that the LS variable is robust. The constriction factor rises from 0.678 to 0.936, which implies that the direct estimation has the greatest participation within the model (Figure 3).

## Results

Table 4 presents the model’s estimates for LS. Columns 1 and 2 show the estimates when the variable LS is used as the dependent variable. On the other hand, columns 3 and 4 show the estimates when the variable LS estimated by SAE is used as the dependent variable.

**TABLE 4:** Determinants of life satisfaction.

	Dependent variable:		Dependent variable:	
	life satisfaction		life satisfaction estimated by SAE	
	(1)	(2)	(3)	(4)
Segregation	0.288**	0.287**	0.233**	0.236**
	(0.124)	(0.126)	(0.0924)	(0.0917)
Lag. Segregation	–	0.0477	–	0.0731
		(0.187)		(0.132)
Log (monetary household income)	0.466***	0.443**	0.398***	0.382***
	(0.171)	(0.172)	(0.121)	(0.128)
% People in public health system	−0.498***	−0.537**	−0.446**	−0.457**
	(0.243)	(0.253)	(0.186)	(0.192)
% People in private health system	−0.263	−0.493	0.0126	−0.0976
	(0.559)	(0.573)	(0.379)	(0.394)
Labor participation rate	−0.747	−0.684	−0.733*	−0.683
	(0.612)	(0.633)	(0.420)	(0.438)
Unemployment rate	−1.697	−2.098*	−1.438*	−1.720*
	(1.212)	(1.239)	(0.862)	(0.887)
% Women	1.324	1.226	1.239	1.189
	(0.999)	(1.006)	(0.785)	(0.790)
Age	−0.136**	−0.144**	−0.122***	−0.128***
	(0.0608)	(0.0618)	(0.0418)	(0.0429)
Age squared	0.00154*	0.00169*	0.00140**	0.00153**
	(0.0008)	(0.0009)	(0.000632)	(0.000661)
% Elderly	−1.100	−1.411	−1.282	−1.547
	(2.037)	(2.127)	(1.518)	(1.653)
10/40 Index	0.000496	−0.000185	−0.000129	−0.00101
	(0.00219)	(0.00213)	(0.00162)	(0.00146)
Constant	3.691	3.838	4.128**	4.368**
	(2.492)	(2.508)	(1.769)	(1.826)
Observations	310	305	310	305
R squared	0.165	0.151	0.261	0.239

Life satisfaction is measured in a scale ranging from 0 to 10. Robust standard errors in parentheses.

***p < 0.01; **p < 0.05; *p < 0.1.

*SAE, small area estimation.*

*Source: Casen survey 2011 and 2013.*

Firstly, it stands out as the main result that the segregation variable has a positive and statistically significant impact (at 5%) on LS. This result is maintained regardless of whether it is estimated with the LS variable (columns 1 and 2) or with the LS variable estimated by SAE (columns 3 and 4). Since socioeconomic segregation can have a dynamic effect on the LS variable, its lag is incorporated into the right side of the equation. When this is done, it is appreciated that the lagged variable does not have a statistically significant effect.

With respect to the other variables incorporated in the model, the results are in line with the previous empirical evidence.^1^ The coefficient associated with the logarithm variable of household monetary income is positive and statistically significant (in three of the four models, it is significant at 1%). This means that the monetary income of the home has a diminishing marginal return on LS. This result is maintained regardless of whether it is estimated with the LS variable or with the LS variable estimated by SAE. With respect to age, a U-shaped relationship is seen. This means that satisfaction with life is greater at the ends of life and reaches a minimum value around the average age of the person. In fact, it is possible to calculate the age at which satisfaction with life is minimized, which in the case of Chile, and according to these estimates, is obtained at 42 years of age. Finally, it should be noted that the four models have a high R squared (between 15 and 26%).

One aspect that may affect the previous estimates has to do with the role of the unobservable factors, which would bias the estimated coefficients.^2^ If these unobservable factors are assumed to be invariant over time, a difference estimate eliminates the problem. Therefore, Table 5 presents the results of estimating the model in differences, with data from the Casen survey 2011 and 2013.

**TABLE 5:** Determinants of life satisfaction (model in differences 2013–2011).

	Dependent variable: Dif. in life satisfaction	Dependent variable: Dif. in life satisfaction estimated by SAE
Segregation	0.322***	0.259***
	(0.123)	(0.0886)
Log (monetary household income)	0.401	0.419**
	(0.255)	(0.175)
% People in public health system	0.138	0.124
	(0.488)	(0.344)
% People in private health system	0.209	−0.183
	(0.773)	(0.519)
Labor participation rate	0.525	0.961**
	(0.631)	(0.467)
Unemployment rate	−2.763*	−2.712***
	(1.520)	(1.006)
% Women	1.622	1.554*
	(1.201)	(0.812)
Age	0.0373	0.0247
	(0.0788)	(0.0494)
Age squared	−0.000608	−0.00032
	(0.00108)	(0.000663)
% Elderly	1.388	0.756
	(2.037)	(1.387)
10/40 Index	−0.0000446	0.00011
	(0.00314)	(0.00219)
Constant	0.218***	0.218***
	(0.0678)	(0.0479)
Observations	305	305
R squared	0.048	0.08

Life satisfaction is measured in a scale ranging from 0 to 10. Robust standard errors in parentheses.

***p < 0.01; **p < 0.05; *p < 0.1.

*SAE, small area estimation.*

*Source: Casen survey 2011 and 2013.*

The results show that the variable socioeconomic “segregation” is, practically, the only variable that remains significant. The coefficient associated with it is positive and statistically significant at 1%. The logarithm of household monetary income has a positive effect on LS but only when the LS variable estimated by SAE is used. And the unemployment rate has a negative effect on the LS of the population.

A final aspect that is evaluated has to do with a supposed persistence that the variable LS may have. That is why an estimation of a first-order autoregressive model is carried out for the variable LS. The results of the estimation for a model with these characteristics are presented in Table 6. Columns 1 and 2 present the estimates when the variable LS is considered as the dependent variable, while columns 3 and 4 show the estimates when the LS variable estimated by the SAE methodology is used. It should be noted that columns 2 and 4 show the estimates when the lagged segregation variable is added as a control.

**TABLE 6:** Determinants of life satisfaction (evaluating persistence).

	Dependent variable:		Dependent variable:	
	life satisfaction		life satisfaction estimated by SAE	
	(1)	(2)	(3)	(4)
Segregation	0.278**	0.291**	0.197**	0.204**
	(0.124)	(0.128)	(0.0922)	(0.0934))
Lag. segregation	−	0.0940	−	0.0576
		(0.196)		(0.140)
Log (monetary household income)	0.346	0.348	0.230	0.228
	(0.226)	(0.232)	(0.166)	(0.175)
% People in public health system	−0.270	−0.318	−0.161	−0.194
	(0.325)	(0.328)	(0.242)	(0.244)
% People in private health system	0.106	−0.135	0.424	0.294
	(0.527)	(0.550)	(0.362)	(0.382)
Labor participation rate	−0.0310	0.0183	−0.0847	−0.0683
	(0.639)	(0.651)	(0.454)	(0.462)
Unemployment rate	−1.952	−2.289*	−1.519*	−1.721*
	(1.213)	(1.254)	(0.878)	(0.918)
% Women	1.504	1.405	1.487*	1.410*
	(1.004)	(1.017)	(0.769)	(0.780)
Age	−0.119*	−0.126**	−0.102**	−0.106**
	(0.0612)	(0.0624)	(0.0417)	(0.0428)
Age squared	0.00129	0.00142	0.00113*	0.00121*
	(0.000887)	(0.000923)	(0.000624)	(0.000654)
% Elderly	−0.503	−0.805	−0.607	−0.793
	(2.067)	(2.167)	(1.564)	(1.640)
10/40 Index	−0.000168	−0.000513	−0.000913	−0.00118
	(0.00216)	(0.00219)	(0.00147)	(0.00146)
Life satisfaction 2011	0.0676	0.0483	−	−
	(0.0537)	(0.0579)		
Life satisfaction 2011 estimated by SAE	−	−	0.167***	0.152***
			(0.0475)	(0.0542)
Observations	310	305	310	305
Constant	3.987	4.190	4.410*	4.636*
	(3.366)	(3.457)	(2.494)	(2.615)
R squared	0.162	0.146	0.279	0.251

Life satisfaction is measured in a scale ranging from 0 to 10. Robust standard errors in parentheses.

***p < 0.01; **p < 0.05; *p < 0.1.

*SAE, small area estimation.*

*Source: Casen survey 2011 and 2013.*

The main result that has been shown is maintained, that is, segregation has a positive and statistically significant effect (at 5%) on the satisfaction with life of the population. The difference that can be seen basically has to do with the effect of the lagged dependent variable. While in models 1 and 2, it is seen that the lagged variable does not have a significant effect on the LS variable, in models 3 and 4, a positive and statistically significant effect is found.

The results indicate that segregation continues to have a positive and significant effect at 1%. Also note that the expected signs and significance for the household income variables, measured in logarithm, and the labor force are maintained (Table 7).

**TABLE 7:** SAE for life satisfaction measured as proportion.

	Model (1)	Model (2)
	Life satisfaction (≥6)	Life satisfaction (≥6)
Number of people in formal employment system	−0.0143***	−0.0104*
	(0.00528)	(0.00530)
SAE poverty rate	−0.179***	−0.243***
	(0.0589)	(0.0643)
% of Rurality	−0.0627**	−0.0221
	(0.0299)	(0.0308)
Region 7 (D = 1)		−0.0426**
		(0.0184)
Region 9 (D = 1)		0.0486**
		(0.0195)
Region 5 (D = 1)		0.0395**
		(0.0169)
Constant	1.305***	1.265***
	(0.0541)	(0.0549)
Observations	324	324
R-squared	0.066	0.118

Standard errors in parentheses.

***p < 0.01; **p < 0.05; *p < 0.1.

Finally, it is worth mentioning that the segregation variable is robust to these considerations. Significance is maintained by changing its nature and by incorporating the SAE methodology to evaluate its robustness, even when we have a variance estimation that is more exact than the previous ones.

A requirement for the arcosenic transformation is that the prediction of the synthetic model must be kept within the real numbers that the transformation supports. By definition, the arcsine of a variable X is real if its values are in the range [0,$\frac{\pi }{2}$]. The prediction values for the LS variable range between 1.03 and 1.22, which guarantees valid results.

In this way, what has been shown, through the different models that have been estimated, is that the socioeconomic segregation variable has a robust, positive, and statistically significant impact on the LS.

## Discussion

We have tested the hypothesis that socioeconomic segregation has a negative effect on individuals’ LS. Several investigations produced by social scientists coming from different backgrounds such as sociology, psychology, and economics have shown the pernicious consequences of segregation on well-being. Consequently, at first sight, we would expect to observe a similar result regarding segregation impact on LS. Notwithstanding, our finding goes in an opposite direction: socioeconomic segregation has a positive effect on LS. Despite the latter may seem as a striking result, there is empirical and theoretical background supporting it. First, similar results have been found about the effects of racial segregation on SWB in the USA regarding the fact that households are happier living among households of the same race. Social capital may be increased as well if people of similar income, needs, and tastes share the same neighborhoods. Segregated households may have a greater sense of belongingness, social cohesion, trust, participation, and mutual support and collective action. Another possible explanation to this result has to do with the acculturation process. For instance, in an integrated neighborhood, people of different socioeconomic backgrounds may face the stress of acculturation, which would have a negative effect on LS. Albeit it is not the same situation that has been studied here, it has been found in literature that immigrants must deal with the stress due to acculturation and rooting with the host country (Urzúa et al., 2019). Hence, our finding has support in literature. However, we are aware about the severe negative effects that segregation imposes to minorities. Additionally, we have to consider the fact that even if segregation has a positive effect on LS, it does not mean that the final effect on well-being will be positive, as both concepts are not the same, as the former is just a constituent part of the latter (Diener, 1984; Samman, 2007; Busseri and Sadava, 2010; Diener et al., 2018; Ruggeri et al., 2020). But the problem seems to be that grown-ups and adolescents will face just disruptive effects because of the dismantling of their social networks, among others, if they are moved to richer neighborhoods. Therefore, any public policy design to reduce segregation should take into account these effects and maybe it should be focused on children; otherwise, it will cause just distress, a reduction of LS, and its positive impact will be almost negligible.

## Data Availability Statement

Publicly available datasets were analyzed in this study. This data can be found here: http://observatorio.ministeriodesarrollosocial.gob.cl/encuesta-casen.

## Author Contributions

RM: econometric analysis, results’ analysis, and interpretation. MV: research question, methodological design, results’ interpretation, and write down the document. DV: econometric analysis and data analysis. All authors contributed to the article and approved the submitted version.

## Conflict of Interest

The authors declare that the research was conducted in the absence of any commercial or financial relationships that could be construed as a potential conflict of interest.

## Footnotes

1 These results should be viewed with caution, as there are potentially other elements that also affect life satisfaction. For example, the role played by unobservables (in terms of individual personality traits) is well documented in the literature. Unfortunately, with the information available, it is not possible to take these types of factors into account. However, the signs for the rest of the explanatory variables incorporated in the model have the expected sign.

2 These unobservables have to do with those existing at the municipality level.
